# Ultra-Sensitive Detection of Chloramphenicol by CdS@NiMoS Nanorods-Based Photoelectrochemical Aptasensor

**DOI:** 10.3390/bios15070454

**Published:** 2025-07-14

**Authors:** Hebin Sun, Yimeng Sun, Tong Qi, Zhenyu Wang, Jianlong Zhao, Lijuan Liang

**Affiliations:** 1 State Key Laboratory of Transducer Technology, Shanghai Institute of Microsystem and Information Technology, Chinese Academy of Sciences, Shanghai 200050, China; 2 School of Materials Science and Engineering, Harbin Institute of Technology, Harbin 150001, China; 3 Center of Materials Science and Optoelectronics Engineering, University of Chinese Academy of Sciences, Beijing 100049, China

**Keywords:** CdS@NiMoS, chloramphenicol, aptsensor, PEC

## Abstract

A novel nanomaterial photoelectrochemical aptamer sensor based on CdS@NiMoS heterojunction nanocomposites was constructed for highly sensitive detection of chloramphenicol (CAP) in antibiotic residues. Through optimization of the material synthesis process, the optimal doping ratio of MoS_2_ to Ni^3+^ (70% MoS_2_ and 10% Ni^3+^) was identified, which significantly enhanced the photogenerated carrier separation efficiency. In thin-film preparation, comparative analysis of four film-forming methods led to the determination of an optimal process with stability. To achieve highly specific CAP detection, the nanocomposite chip was integrated with nucleic acid aptamer biorecognition elements within a standard three-electrode detection system. Experimental results demonstrated a linear response (R^2^ = 0.998) in the 0.1–2 μM concentration range, with a detection limit of 3.69 nM (3σ/S).

## 1. Introduction

Chloramphenicol (CAP), a broad-spectrum antibiotic originally from Streptomyces venezuelae, was once widely used in clinical treatment due to its significant inhibitory effect on Gram-positive, Gram-negative, and some anaerobic bacteria [1,2]. However, subsequent studies found that the drug may cause serious hematological toxic reactions such as aplastic anemia and bone marrow suppression [1,3,4]. Currently, China, the United States, and other countries have strictly restricted its use in clinical treatment and as a food additive [5,6,7]. Nevertheless, with the growing problem of antibiotic abuse worldwide, the demand for CAP residue testing continues to grow, with the development of highly sensitive detection techniques for CAP being particularly important [8,9,10].

In recent years, a series of advanced analytical techniques have been developed for antibiotic detection, including colorimetric [11,12], fluorescence [13,14], surface plasmon resonance [15,16], electrochemical [17,18], chemiluminescence [19,20], and photoelectrochemical (PEC) methods [21,22]. Among these, photoelectrochemical aptasensors have emerged as a particularly promising analytical platform in the field of detection science, owing to their exceptional advantages, including high sensitivity, low background noise, rapid response, outstanding selectivity, and excellent cost-effectiveness [23,24,25]. PEC-based detection operates on the principle of photoexcitation in semiconductor materials, where electron–hole pairs are generated upon light irradiation, producing measurable photocurrent signals under an applied electric field [26]. This mechanism enables highly sensitive and selective target detection. Furthermore, nucleic acid aptamers, serving as recognition elements, offer significant benefits such as ease of preparation, low cost, flexible chemical modification, high stability, and excellent target-binding specificity [27]. By strategically designing photosensitive materials and optimizing probe molecule immobilization, PEC aptamer sensors can achieve selective identification of target analytes even in complex matrices. As a result, this technology has demonstrated tremendous potential in environmental monitoring, food safety, and clinical diagnostics [28,29,30].

Numerous studies have demonstrated the promising potential of the PEC-aptsensor for trace antibiotic detection. For instance, a representative study developed a novel PEC biosensor using nitrogen-doped graphene quantum dots (N-GQDs) as a visible light-responsive material. Experimental results revealed a positive correlation between the photocurrent response and CAP concentration [31]. Another approach employed graphene quantum dots/titanium dioxide nanotubes (GQDs/TiO_2_ NTs) nanocomposites integrated with PEC aptamer sensing technology, achieving highly efficient CAP detection with both a broad linear detection range and remarkable sensitivity [32]. Most recently, Wu et al. introduced an innovative ITO/p-W(Fe)O_3_-cDNA/apt aptasensor for CAP detection. The results demonstrated linear correlation (R^2^ = 0.99) between photocurrent reduction and CAP concentration across a wide detection range (1.0 pM–10.0 nM) [33].

As a typical II-VI semiconductor, cadmium sulfide (CdS) can be solvothermally modulated in crystallographic orientation to form multilevel microstructures ranging from nanorods to hexagonal prisms. Its unique carrier mobility and narrow bandgap of 2.4 eV endow the material with excellent photoresponsivity [34,35]. Molybdenum disulfide (MoS_2_) has become a hotspot in the study of transition metal sulfide compounds by virtue of its unique 1.75–2.1 eV tunable bandgap and interlayer quantum-limited-domain effect, which exhibits a significantly enhanced carrier separation efficiency in the visible region [36]. While both sulfides have demonstrated exceptional performance in photocatalytic antibiotic degradation, their integration with molecular recognition elements remains largely unexplored. Particularly, the development of aptamer-functionalized PEC biosensors for CAP detection has rarely been reported [37,38,39].

Our report introduces a new type of efficient CdS@NiMoS nanocomposite with a heterojunction structure, which exhibits significantly enhanced light absorption and charge separation efficiency. On this basis, we used aptamers as specific biorecognition elements to develop high-performance photoelectrochemical aptamer sensors. The experimental results show that the sensor exhibits excellent analytical performance in the field of CAP trace detection, providing a sensitive, rapid, and reliable new analytical platform for CAP environmental pollutant monitoring and food safety testing.

## 2. Materials and Methods

### 2.1. Materials and Apparatus

Chloramphenicol (CAP), tetracycline hydrochloride (TETRA), kanamycin (KANA), and gentamycin sulfate (GENTA) were purchased from Shanghai Aladdin Biochemical Technology Co., Ltd. (Shanghai, China); streptomycin sulfate (STREP) was purchased from Tokyo Chemical Industry Co., Ltd. (Tokyo, Japan) The aptamer for CAP was purchased from Sangon Biotech (Shanghai, China). Cd(NO_3_)_2_-4H_2_O, Na(DDTC), NaMoO_4_-2H_2_O, CN_2_H_4_S, and Ni(NO_3_)_2_-6H_2_O were purchased from Shanghai Aladdin Biochemical Technology Co. (Shanghai, China) The CAP-specific aptamer (5′-SH C6-AGC AGC ACA GAG GTC AGA TGC ACT CGG ACC CCA TTC TCC TTC CAT CCC TCA TCC GTC CAC CCT ATG CGT GCT ACC GTG AA-3′), DNA buffer, and phosphate-buffered saline (PBS) were obtained from Sangon Biotech (Shanghai, China). ITO-coated glass substrates were provided by Liaoning Youxuan New Energy Technology Co., Ltd. (Dalian, China) All aqueous solutions were prepared using autoclaved, double-distilled deionized water (18.2 MΩ·cm resistivity) to minimize potential contamination. All chemical reagents were of analytical grade unless otherwise specified.

### 2.2. CdS@NiMoS Nanorods Synthesis

Cd(NO_3_)_2_-4H_2_O at 1.5 mM and Na(DDTC) at 30 mM were sonicated for 10 min. Cd(DDTC)_2_ precipitates were produced after thorough stirring. After washing, filtration, and vacuum drying, the precursor was obtained. Mixed 2 g Cd(DDTC)_2_ with 70 mL ethylenediamine in a 100 mL Teflon reactor. Heated at 180 °C for 24 h in an oven. The CdS nanorods were obtained by washing once with deionized water and three times with ethanol, followed by aspiration. Dispersed the CdS nanorods in deionized water (Beaker A) and dissolved NaMoO_4_·2H_2_O, CN_2_H_4_S, and Ni(NO_3_)_2_·6H_2_O in deionized water (Beaker B). Sonicated both solutions for 10 min, then transferred them to a 50 mL reactor and reacted at 200 °C for 24 h. The final product was washed with deionized water and ethanol, followed by suction filtration and vacuum drying at 60 °C for 5 h to obtain CdS@NiMoS nanorods.

### 2.3. Fabrication of PEC-Aptasensor

The ITO glass was cleaned by ultrasonication with ethanol/NaOH solution (1:1 volume ratio, NaOH concentration is 0.1 M), acetone, and deionized water (30 min each time) and then blown dry by nitrogen for use. CdS@NiMoS nanorods were mixed with iodine powder (mass ratio of 1:2) in 30 μL of Nafion (1 mg/100 μL) solution and sonicated to form a homogeneous slurry. A two-electrode system was used (spacing of 1 cm) to obtain dense composite electrodes. The two-electrode system was plated at a constant voltage of 10 V for 5 min and then dried (120 °C for 30 min) to obtain a dense composite electrode. A total of 50 μL of aptamer solution (0.3 μM) was coated evenly on the electrode surface, and the aptamer-CdS@NiMoS chip was incubated at 25 °C for 6 h to complete the preparation of the aptamer-CdS@NiMoS chip. The CAP solution was added dropwise on the surface of the modified chip and incubated for 6 h under the same environment. Finally, the CAP-aptamer-CdS@NiMoS detection chip was obtained. The morphology of CdS@NiMoS nanorods on ITO glass was characterized by scanning electron microscopy (SEM) and transmission electron microscopy (TEM) using ethanol as the dispersant.

### 2.4. Preparation of Nanofilm

Ethanol drop-casting: 100 μL of the target ethanol dispersion was quantitatively transferred with a pipette and uniformly coated on the ITO surface by drop coating, and then gradient baking was performed under an infrared drying lamp (power 250 W, wavelength 2.5–5 μm) for 5 min. PVDF method: 5.0 mg of sample was weighed and mixed with 10 μL of 20 wt% polyvinylidene fluoride (PVDF), 190 μL of methanol, and 1 mL of N-methyl-2-pyrrolidone (NMP, 20 mg/mL). The mixture was then subjected to ultrasonication (300 W, 40 kHz) for 1 h. Then, the sample was self-assembled into a film under constant-temperature infrared lamp irradiation. Nafion drop-casting: Nafion-D-520 proton exchange resin was dissolved in a mixed solvent of deionized water and 1-propanol at a concentration of 5% (*w*/*w*), 500 μL of Nafion dispersion was ultrasonically treated to form a uniform slurry, and dried under an infrared lamp. Electroplating: A mixture of 20 mg of sample, 40 mg of purified iodine, and 40 mL of acetone was coated on the surface and ultrasonicated for 1 h. After standing for 10 min, the supernatant was taken out and electroplated at 10 V for 10 min, and then placed in an oven for low-temperature annealing at 120 °C for 40 min.

### 2.5. PEC Detection

Photoelectrochemical measurements were performed using an electrochemical workstation produced by Zahner. A 500 W ultraviolet xenon lamp was used as the excitation light source, with monochromatic light of specific wavelengths selected via a grating-based monochromator to irradiate the sample. The constant bias applied to the working electrode was −0.25 V (vs. Ag/AgCl reference electrode). A three-electrode system was employed to perform detailed measurements across a low-concentration range (0.1–2 μM). All experiments were carried out at a constant temperature of 25 ± 0.5 °C. The electrochemical workstation was controlled by Thales software 5.7.0, and the current–time (i–t) curve was collected in real time, and data was processed.

## 3. Results

### 3.1. Sensing Mechanism

**Figure 1:**
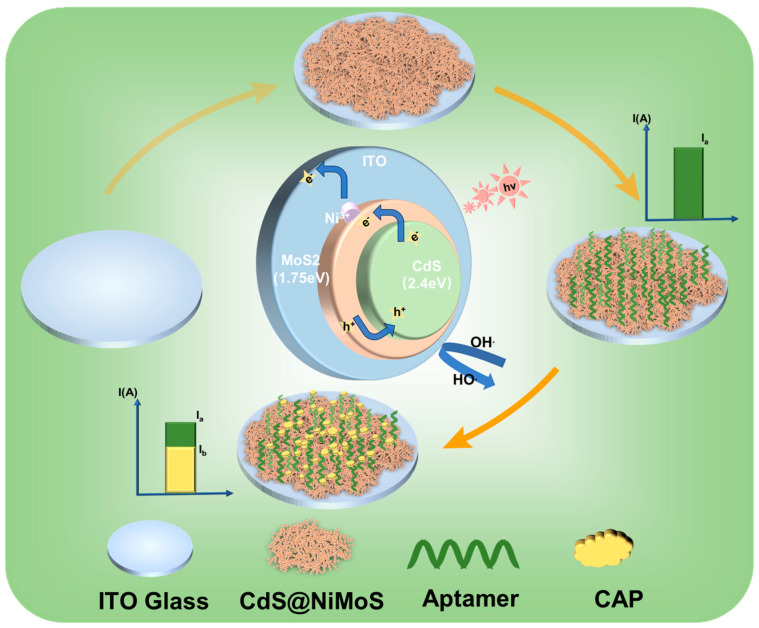
Principle of the PEC-aptasensor for the detection of CAP.

The experimental strategy is shown in Figure 1. Using CAP as the model antibiotic, we utilize ITO glass as the conductive substrate and electroplate the photosensitive material onto the ITO to form a film. The selected aptamer–ferrocene complex (Apt-Fc) is incubated on the surface of the film-formed material. The aptamer serves as the CAP recognition and capture reagent, while the ferrocene part is used as an electron donor to reach peak photocurrent (I_a_). Conversely, when CAP is added dropwise to the electrode, the nucleic acid aptamer will capture the CAP in solution; the specific binding of the nucleic acid aptamer to CAP results in a reduction in the photocurrent of the photosensitive material. The reason for this is that the binding of CAP to the aptamer further reduces the affinity of the aptamer to the photosensitive material, making it easy for the electron donor to detach from the electrode surface, and the current I_a_ decreases to the current I_b_, and therefore the difference in current can be used to determine the antibiotic content.

### 3.2. Optimization of Experimental Conditions

**Figure 2:**
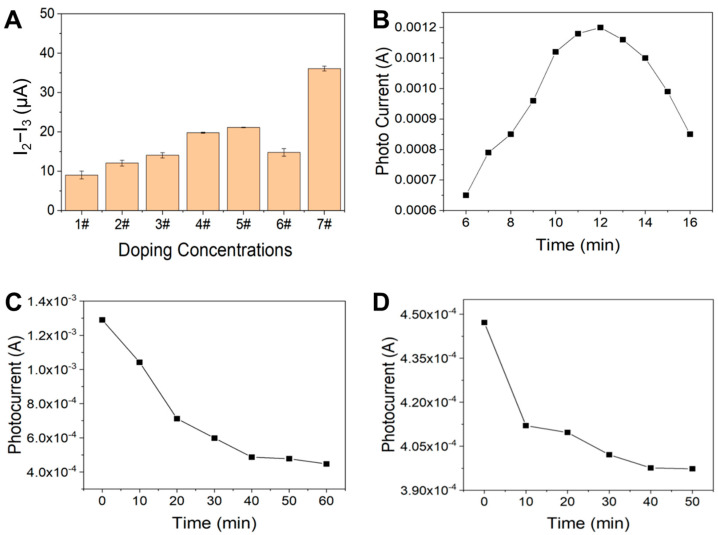
(**A**) Effect of MoS_2_/NiMoS content on the photocurrent response for CAP detection 1#: 100%CdS; 2#: CdS@10%MoS_2_; 3#: CdS@30%MoS_2_; 4#: CdS@50% MoS_2_; 5#: CdS@70% MoS_2_; 6#: CdS@90% MoS_2_; 7#: CdS@70% MoS_2_@10%Ni^3+^. (**B**) Effect of nano photoelectric film-forming plating time on photocurrent. (**C**) Photocurrent response at different times after incubation of aptamer (0–60 min); (**D**) CAP immobilization time on PEC response (0–50 min).

To understand the extent of the difference in material composition at each step, a series of compositional optimizations of chemical synthesis and bioincubation processes were performed. Here, we define the photocurrent current of ITO itself as I_0_, the photocurrent of ITO + CdS@NiMoS as I_1_, the photocurrent of ITO + CdS@NiMoS + Apt as I_2_, and the photocurrent of ITO + CdS@NiMoS + Apt + CAP as I_3_. As shown in Figure 2A, the formation of heterojunctions after the addition of MoS_2_ to CdS results in a gradual increase in the photocurrent difference, which reaches a maximum at 70% of the composite concentration. The addition of 10% Ni^3+^ on this basis can further improve the mobility and reduce the compounding of electron–hole pairs, completing the optimization of the chemical synthesis composition of photosensitive material.

To enable the PEC-aptasensor to detect CAP level more accurately, we optimized three key parameters in our experiments: plating time, aptamer incubation time, and antibiotic fixation time. First, since the plating time of the photosensitive material directly affects the substrate photocurrent intensity, we examined the electric current response in the range of 6–16 min. As shown in Figure 2B, the photocurrent signals peaked when the CdS@NiMoS nanomaterials were plated for 12 min, indicating that the optimal photoactive interface was formed at this time. Secondly, for the assembly process of aptamer–ferrocene complexes, it was found by time gradient experiments that the photocurrent signals stabilized after 40 min with the prolongation of the incubation time (Figure 2C), suggesting that the assembly of the complexes reached an equilibrium state. Finally, to confirm the sufficient binding of CAP to the aptamer, we monitored the immobilization kinetics during 0–50 min. As shown in Figure 2D, the slope of the response curve decreased significantly after 40 min, indicating that the binding reaction was close to saturation. Based on the above experimental results, we finally determined the optimal conditions as 12 min for CdS@NiMoS plating, 40 min for aptamer incubation, and 40 min for CAP immobilization.

### 3.3. Characterization of CdS@NiMoS Nanorods on ITO Glass

**Figure 3:**
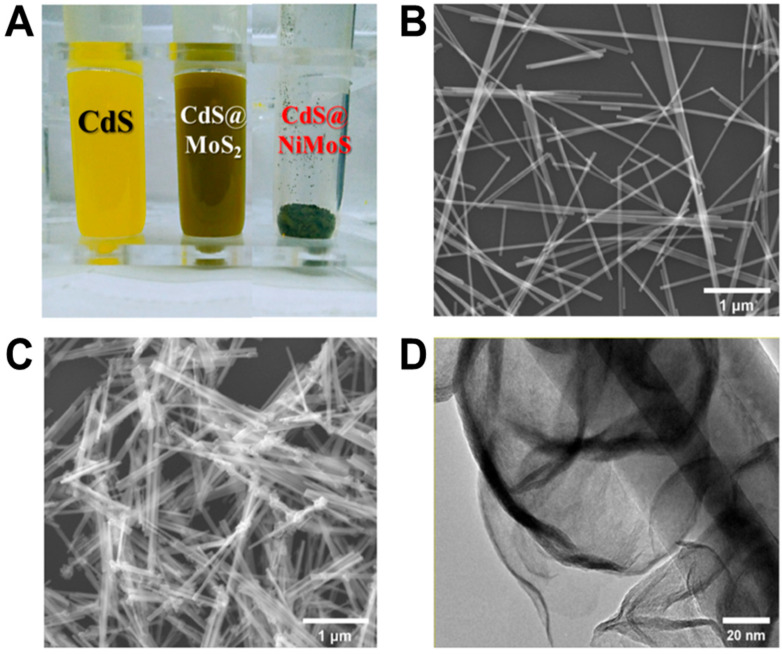
(**A**) The physical diagram of the fabricated photosensitive nanomaterial; (**B**,**C**) SEM images of CdS nanorods and CdS@NiMoS nanorods, respectively; (**D**) TEM image of CdS@NiMoS nanorods.

The fabricated photosensitive nanomaterial is presented in Figure 3A. As observed, the CdS nanorods dispersed in ethanol exhibit a homogeneous bright yellow coloration, whereas the CdS@MoS_2_ composite solution appears gray-green, and the CdS@NiMoS-doped composite solution presents a dark green hue. The morphological features of the CdS@NiMoS nanorods grown on ITO glass were investigated using scanning electron microscopy (SEM) and transmission electron microscopy (TEM). As shown in Figure 3B,C, the MoS_2_ nanosheets, along with the doped transition metal Ni, form a spiral-coiled structure enveloping the outer layer of the CdS nanorods. This architecture not only maximizes the specific surface area but also effectively modulates the bandgap of the composite material. Furthermore, the bandgap can broaden the light absorption range of the material within the visible spectrum, thereby enhancing its photoelectric conversion efficiency. The TEM image in Figure 3D reveals the NiMoS nanosheets spiraling around the CdS nanorods, providing evidence for the successful synthesis of the Ni-doped CdS@MoS_2_ structure.

### 3.4. Exploring Film Formation

**Figure 4:**
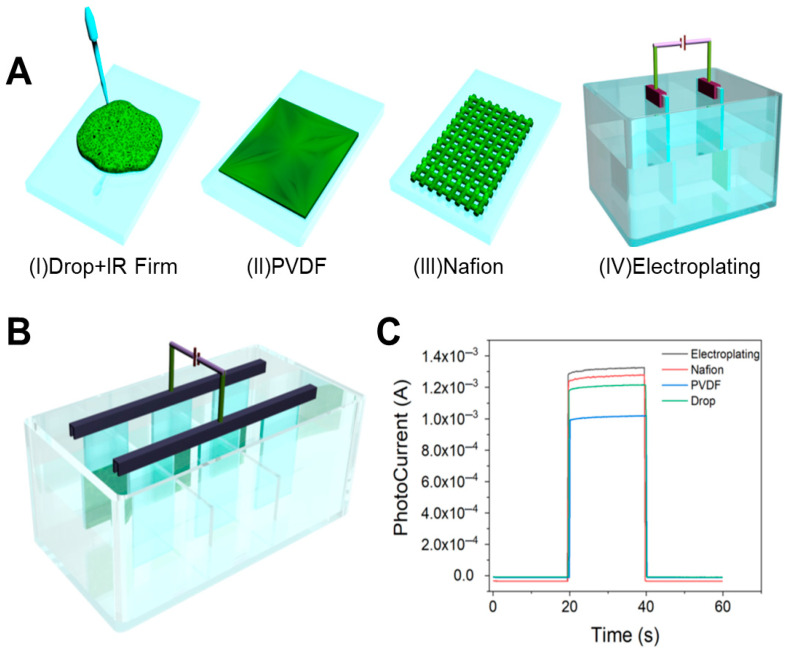
(**A**) Exploration of film formation methods: (I) drip coating method; (II) poly(1,1-difluoroethylene) (PVDF) coating method; (III) Nafion coating method; (IV) electroplating method. (**B**) Optimized preparation of PEC sensors by electroplating method. (**C**) CHOP diagram of photocurrent response of four film-forming methods.

Film formation optimization of photosensitive nanomaterials is essential to achieve stable nanofilms with consistent performance. Hence, we explored four film-forming techniques for the photosensitive nanomaterial, as illustrated in Figure 4A. The drop-coating method resulted in inadequate film formation, prone to peeling off during testing and dispersing in the solution. Similarly, the PVDF-based method could not form a uniform coating even after ultrasonic treatment. The Nafion method, while advantageous due to its ease of application, adjustable concentration, and rapid processing time, suffered from non-uniform film deposition, leading to detachment in liquid environments over extended periods. The electroplating method involved dissolving the material in acetone and iodine solution for double electrode plating. Although resulting in a thin, uniform coating resistant to detachment, this method is time-consuming.

To avoid this problem, Figure 4B demonstrates our innovative redesign of the conventional reaction cell, where strategic partitioning created three independent reaction zones. By integrating this modified cell with a multi-channel electrochemical workstation, we achieved synchronous electrochemical deposition across all compartments. This optimization enhanced sample preparation throughput from one sample per 10 min to nine samples per 10 min, representing a 9-fold increase in experimental efficiency. Comparative analysis of the photocurrent response characteristics is shown in Figure 4C, and the performance is as follows: electroplating > Nafion drop-casting > ethanol drop-casting > PVDF method.

### 3.5. Detection Feasibility Assay

**Figure 5:**
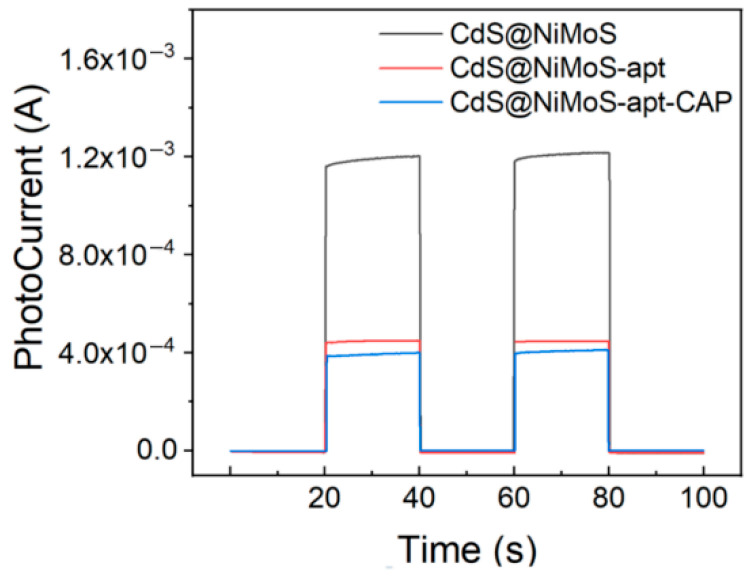
PEC response of different electrodes in 0.1 M PBS containing 0.1 M NaSO_3_.

The photocurrent responses of different electrodes in 0.1 M PBS containing 0.1 M NaSO_3_ are shown in Figure 5. The CdS@NiMoS nanorod-modified electrode prepared by electrodeposition exhibits significantly enhanced and stable photocurrent generation. This phenomenon primarily originates from two mechanisms: (1) the transition metal sulfide coating effectively promotes the separation of photogenerated electron–hole pairs while substantially suppressing their recombination; (2) the iodide-assisted electrodeposition process enables robust bonding between the photosensitive material and ITO substrate, ensuring structural stability of the modified layer. During the aptamer modification stage, the ferrocene groups establish efficient electron transfer channels with the photosensitive material layer, significantly improving the electron conduction efficiency of the system and consequently enhancing the photocurrent intensity. After CAP introduction, the specific binding between aptamers and target molecules weakens the electronic coupling at the ferrocene/photosensitive material interface, obstructing the electron transport pathway and ultimately leading to reduced photocurrent signals.

### 3.6. Detection Performances

**Figure 6:**
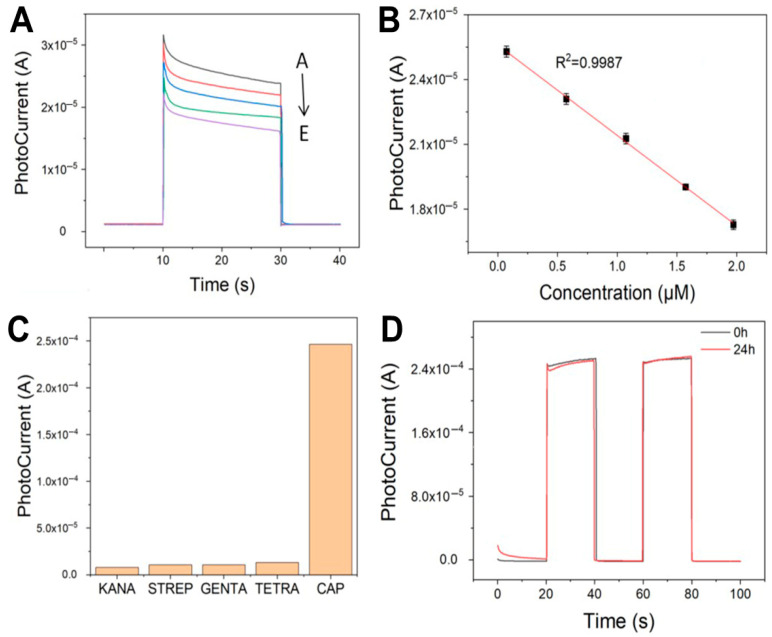
(**A**) Photocurrent response of the fabricated PEC-aptasensor to varying concentrations of CAP (A–E: 0.1, 0.5, 1, 1.5, and 2 μM). (**B**) The linear relationship between photocurrent and CAP concentration. (**C**) Selectivity measurement of PEC-aptasensor. (**D**) Stability measurement of PEC-aptasensor.

The PEC-aptasensor was measured with CAP concentrations ranging from 0.1 to 2 μM with 0.5 mM increments (Figure 6A). The linearity between photocurrent intensity and CAP concentration (correlation coefficient, 0.9987; limit of detection, 3.69 nM) in Figure 6B. In terms of detection sensitivity, the proposed method demonstrates superior performance compared to previously reported photoelectrochemical immunosensors based on thioglycolic acid-capped CdS quantum dots [40], as well as other aptamer-based sensors for CAP detection [41]. However, when benchmarked against certain ultra-high-sensitivity studies [42,43], the detection limit of this approach still exhibits room for improvement. This discrepancy may be attributed to differences in the selection of photoactive materials or the extent of optimization in signal amplification strategies. Regarding the application range, the established linear detection range of 0.1–2 μM is well-suited for routine monitoring of CAP residues in environmental water and food samples [44,45]. Notably, while the upper detection limit of this method is lower than that of molecularly imprinted polymer (MIP)-based PEC sensors [46], it offers enhanced performance in the low-concentration range. This characteristic renders the method particularly advantageous for the precise quantification of trace-level CAP in complex matrices such as environmental media and food samples. Consequently, the proposed sensor holds significant promise for applications requiring high sensitivity in trace CAP detection.

To assess specificity, the PEC-aptasensor was tested against other antibiotics, including KANA, STREP, GENTA, and TETRA. As shown in Figure 6C, the photocurrent response for CAP (2.47 × 10^−4^ A) was significantly higher than that for other antibiotics. In contrast to traditional antibody-based immunosensors, aptamers offer tunable recognition performance through precise sequence design while maintaining high specificity. Moreover, they exhibit excellent stability, retaining activity even after prolonged storage at room temperature, while imposing fewer application constraints.

Furthermore, the long-term stability of the PEC-aptasensor has been validated by I-T curve measurements after 24 h of CAP incubation. The photocurrent remained highly consistent (2.45 × 10^−4^ A), with less than 2% deviation from the initial value, as shown in Figure 6D. This optimized scheme not only ensures the molecular recognition efficiency but also achieves the highest photoelectric conversion performance, providing a reliable sensing platform for CAP detection. Based on the above results, future research directions will focus on enhancing the assay’s sensitivity (potentially reaching the pM level) and expanding its detection range through the implementation of signal amplification strategies.

## 4. Conclusions

In this study, we successfully fabricated CdS@NiMoS nanorod photosensitive electrodes using the PEC method with Nafion as the electroplating solution. The optimized CdS@NiMoS nanorod photosensitive material demonstrated remarkable photoelectrochemical properties, including a high specific surface area and exceptional photoconversion efficiency, as evidenced by its photocurrent response. Through investigation of film formation methods and optimization of incubation time at each step using side-by-side plating techniques, we achieved sensitive detection of CAP with a detection limit of 3.69 nM and excellent linearity in the range of 0.1–2 μM. The developed antibiotic detection method exhibits several advantageous features, including low background current, rapid response time, and high sensitivity, suggesting promising potential for applications in aptamer-based biosensors and small molecule detection platforms.

## Data Availability

The original contributions presented in this study are included in the article. Further inquiries can be directed to the corresponding author.
